# Porous Colorimetric Microneedles for Minimally Invasive Rapid Glucose Sampling and Sensing in Skin Interstitial Fluid

**DOI:** 10.3390/bios13050537

**Published:** 2023-05-10

**Authors:** Qingya Zeng, Mengxin Xu, Weilun Hu, Wenyu Cao, Yujie Zhan, Yuxin Zhang, Qingqing Wang, Tao Ma

**Affiliations:** 1 School of Pharmacy, Bengbu Medical College, Bengbu 233030, China; 2 School of Laboratory Medicine, Bengbu Medical College, Bengbu 233030, China; 3 Anhui Engineering Technology Research Center of Biochemical Pharmaceutical, Bengbu Medical College, Bengbu 233030, China

**Keywords:** porous microneedle, interstitial fluid, glucose, colorimetric detection, diabetes

## Abstract

Though monitoring blood glucose (BG) is indispensable for regulating diabetes, the frequent pricking of the finger by the commonly used fingertip blood collection causes discomfort and poses an infection risk. Since glucose levels in skin interstitial fluid (ISF) correlate with blood glucose levels, monitoring glucose in the skin ISF can be a viable alternative. With this rationale, the present study developed a biocompatible porous microneedle capable of rapid sampling, sensing, and glucose analysis in ISF in a minimally invasive manner, which can improve patient compliance and detection efficiency. The microneedles contain glucose oxidase (GOx) and horseradish peroxidase (HRP), and a colorimetric sensing layer containing 3,3′,5,5′-tetramethylbenzidine (TMB) is on the back of the microneedles. After penetrating rat skin, porous microneedles harvest ISF rapidly and smoothly via capillary action, triggering the production of hydrogen peroxide (H_2_O_2_) from glucose. In the presence of H_2_O_2_, HRP reacts with TMB contained in the filter paper on the back of microneedles, causing an easily visible color shift. Further, a smartphone analysis of the images quickly quantifies glucose levels in the 50–400 mg/dL range using the correlation between color intensity and glucose concentration. The developed microneedle-based sensing technique with minimally invasive sampling will have great implications for point-of-care clinical diagnosis and diabetic health management.

## 1. Introduction

Diabetes is a group of metabolic diseases characterized by hyperglycemia resulting from defects in insulin secretion, action, or both [1]. The stringent monitoring of blood glucose levels can significantly enhance the quality of diabetes treatment while lowering the risk of complications [2,3]. According to the guidelines, routine monitoring must be carried out at least four times a day [4]. The monitoring by a blood glucose meter after pricking the finger remains the most commonly used method in clinics and at home, but it damages the skin and poses an infection risk. Additionally, it reduces patient compliance due to the pain caused by the needle prick, making it unsuitable for long-term continuous blood glucose monitoring [5]. Thus, it is highly desirable to find a new way to measure glucose in body fluids as a supplement to blood glucose measurement.

In recent years, skin interstitial fluid (ISF) has been considered an alternative source of biomarkers [6], due to the reliable correlation between blood glucose levels and the level found in ISF [7,8]. Existing ISF sampling methods such as suction blistering, microdialysis, and laser perforation are time-consuming to apply, can damage the surrounding skin tissue, and require skilled personnel to operate specialized equipment [9]. Microneedles (MNs), usually less than 1 mm long, are widely used to deliver therapeutic drugs and extract biomarkers from ISF. They are minimally invasive, painless, cause little damage to skin tissue, do not irritate subcutaneous nerves and blood vessels, heal quickly, and are simple to use [10,11,12,13]. So far, solid MNs, hydrogel MNs, hollow MNs, and porous MNs have been developed for the sampling of ISF. Solid MNs usually consist of conductive metal materials as sensing electrodes, which pose a potential health risk to patients due to the poor biocompatibility and non-degradable nature of the materials used in these devices [14,15]. Although the hydrogel MN preparation process is simple, the amount of ISF collected by swelling capacity is low, necessitating additional processing steps from collection to release [12,16,17,18]. The hollow MN device is usually expensive and requires complex manufacturing processes such as laser and etching, which greatly limits their development [8,19,20,21]. Compared to other MNs, porous MNs have attracted considerable attention due to their excellent biocompatibility and unique pore structure. They contain a large number of continuously interconnected nano- or micron-sized pores which, after penetrating the skin, swiftly sample large quantities of biofluids by capillary action and deliver them directly to the analysis device for easy and effective diagnosis [22,23,24].

In previous studies on porous MNs for glucose detection in ISF, Beomjoon Kim et al. prepared flexible polydimethylsiloxane (PDMS) porous MNs by salt leaching with hyaluronic acid (HA) coating [23]. However, when applied to mice, PDMS is a hydrophobic material that, instead of using capillary forces, results in a long application process. In addition, poly(lactic-co-glycolic) acid (PLGA) porous MNs were prepared to obtain high-pressure (6 MPa) compression at 238 °C, also using salt leaching methods for pore formation, and the investigators have only validated the use of the device in vitro [25]. Furthermore, polylactic acid (PLA) microspheres can form interconnected micropores inside the MNs using a heat treatment bonding method [26]. However, PLA heating requires a temperature of 180 °C, and the toxic organic solvent dichloromethane is also used in the emulsification process. Combining transdermal biosensing with porous MNs can break through the skin barrier to achieve transdermal ion conduction [27]. Modification of the porous MNs, such as nickel-gold plating or filling with charged hydrogels, can reduce transdermal resistance and efficiently sense ISF [28,29]. The extracted ISF needs to be measured by electrochemical methods or recovered for subsequent analysis. These porous MNs are usually demanding in terms of preparation conditions or time-consuming in application, limiting their clinical use and application in wearable medical devices.

In this study, a colorimetric sensor was fabricated by a simple and low-cost manufacturing process integrating enzymes and chromogenic agents on porous MNs. Porous MNs are made from photopolymer materials containing glucose oxidase (GOx), which catalyzes the extraction of glucose from the MN to produce hydrogen peroxide (H_2_O_2_). In the presence of H_2_O_2_, horseradish peroxidase (HRP) catalyzes the oxidation of the 3,3′,5,5′-tetramethylbenzidine (TMB) contained in the filter paper base, causing an immediate color change of the sensing layer, with high selectivity. The white appearance of the porous MNs as well as the white filter paper on the back both enhance the color contrast, allowing the test information to be easily observed by the naked eye only. In addition to visual observations, the photographs can be easily analyzed on a smartphone to assess glucose concentration by the intensity of the color change of the substrate.

## 2. Materials and Methods

### 2.1. Materials

Glycidyl methacrylate (GMA, ≥97%) and polyethylene glycol (PEG) were procured from Sigma-Aldrich (St. Louis, MO, USA). Filter paper grade 1 was sourced from Whatman (Maidstone, Kent, UK). Trimethylolpropane trimethacrylate (TRIM), triethylene glycol dimethacrylate (TEGDMA), Irgacure 184 (≥98%), and 2-methoxyethanol (≥99%) were purchased from Aladdin (Shanghai, China). Glucose oxidase (GOx, 258 U/mg), horseradish peroxidase (HRP, ≥300 U/mg), 3,3′,5,5′- tetramethylbenzidine (TMB), glucose content assay kit, and agar powder were bought from Solarbio (Beijing, China). Wuhan Procell Life Science and Technology Co., Ltd., provided the NCTC clone 929 cells (L929). All other reagents used were of analytical grade, and all solutions were prepared with ultrapure water (Milli-Q water purification system).

Sprague–Dawley (SD) male rats (weight: 160–180 g) were obtained from Jinan Pengyue Laboratory Animal Breeding Co., Ltd. (Jinan, China) (license no: SCXK(LU)20190003). All animal studies were strictly in compliance with the animal ethical standards and usage committee regulations of Bengbu Medical College.

### 2.2. Fabrication of Porous Microneedles

**Figure 1:**
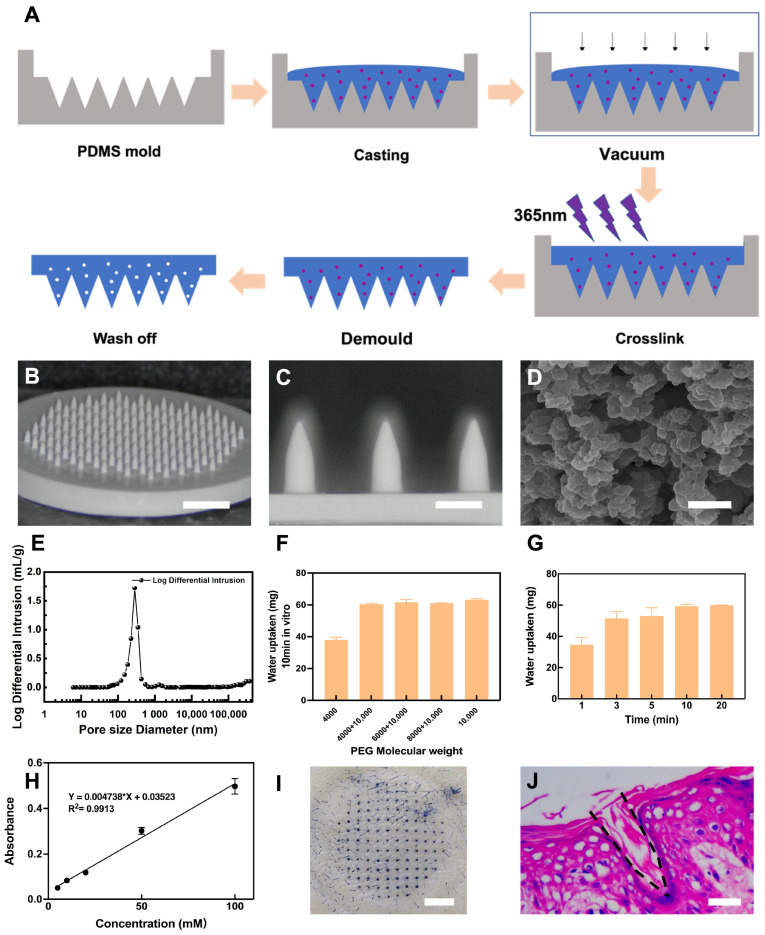
Characterization results of porous MNs. (**A**) Schematic representation of the fabrication process of porous MNs. (**B**) Image of porous MNs. Scale bar: 3 mm. (**C**) Partially magnified image of porous MNs. Scale bar: 500 μm. (**D**) SEM image of porous MNs (PEG molecular weight of 4000 mixed with 10,000 (1:1, *v*/*v*) Scale bar: 1 μm. (**E**) The pore size distribution of porous MNs. (**F**) Absorption behavior of MNs prepared with different molecular weights of PEG in agarose gel (n = 3). (**G**) Effect of porous MN absorption on agarose gels at different time points (n = 3). (**H**) Calibration curve showing the relationship between glucose concentration and absorbance (n = 3). (**I**) Results of a puncture study using MNs on rat skin. Scale bar: 3 mm. (**J**) H&E staining results of skins at the inserted site of the rat. Scale bar: 50 μm.

Using the poly(glycidyl methacrylate) (PGMA) monolithic column preparation method, PEG was used as the porogen [22]. It was then mixed with a GMA monomer for the polymerization reaction assisted by a crosslinker and photoinitiator and finally injected into a PDMS mold for photopolymerization to produce porous MNs. PEG was dissolved in 2-methoxyethanol in a water bath at 50 °C until clear. Irgacure 184 (1 wt% of monomer) was added as the photoinitiator to the homogenous mixture of GMA, TRIM, and TEGDMA. The mixture of monomer solution and porogen solution (1:1, *v*/*v*) was injected into the PDMS mold under a vacuum to ensure that the mixture solution entered the cavity of the mold. After 10 min of UV curing at 365 nm, the MN was removed from the mold and immersed in methanol/water (1:1, *v*/*v*) for 24 h, as depicted in Figure 1A. For the preparation of porous MNs, the following molecular weights of porogen PEG were chosen: pure 4000, blended 4000 and 10,000 (1:1, *v*/*v*), blended 6000 and 10,000 (1:1, *v*/*v*), blended 8000 and 10,000 (1:1, *v*/*v*), and pure 10,000.

### 2.3. Fabrication of Porous Microneedle Sensor

Porous MNs were modified to perform the glucose assay by immobilizing the enzymes on MNs. The prepared MNs were immersed in an adequate mixture of GOx and HRP (120 U/mL, 30 U/mL dissolved in pH 7.2–7.5 PBS) for 10 min (with 1 min sonication) then removed and dried at 37 °C for usage. The detection area was patterned as a 4 mm diameter circle. The circular filter paper was soaked in TMB (3 mM dissolved in anhydrous ethanol) chromogenic agent for 10 min and then removed and dried at room temperature for 30 min. It was then attached to the back of the previously prepared enzyme-containing MNs using a 1.5 cm diameter transparent circular PET sticker to obtain the porous MN sensor. The analytes absorbed by the porous MNs reacted by capillary action and were then transferred to the sensing layer for color development.

### 2.4. Characterization of Porous Microneedles

#### 2.4.1. Physical Characterization of Porous Microneedles

The morphological appearance of the porous MNs was captured using a light microscope and a scanning electron microscope (Thermo Fisher QUANTA 250FEG, Waltham, MA, USA). A mercury porosimeter (Micromeritics AutoPore V 9620, Norcross, GA, USA) was used to measure the mean pore size inside the porous MN.

#### 2.4.2. In Vitro Glucose Extraction and Detection

Porous MNs were inserted into a 1.4% agarose skin model and held for several minutes before being withdrawn, and the difference in mass before and after weighing was recorded as the amount of water uptake.

Porous MNs were inserted into 1.4% agarose skin models containing different concentrations of glucose for 10 min before being removed into 50 mL centrifuge tubes and followed by the addition of 1.5 mL of pure water. The liquid in the tube was recovered after shaking for 1 h, and the absorbance was measured at 505 nm by a glucose assay kit.

#### 2.4.3. Penetration through Rat Skin

For the in vitro puncture performance assay, pre-stripped SD rats’ skin was stabbed with porous MNs for 5 min, removed, and stained with 0.4% trypan blue. The residual dye was wiped away, and the number of pores on the skin was observed under the microscope. In vivo, puncture performance was evaluated by pressing porous MNs onto the back of pre-shaved anesthetized SD rats for 5 min and then removing it. Thereafter, the perforated skin was cut off, fixed in 4% paraformaldehyde, embedded in paraffin, sectioned, stained with hematoxylin and eosin (H&E), and examined under a light microscope.

#### 2.4.4. Protein Retention Test

To investigate the retention of immobilized enzymes soaked in porous MNs during the application, a fluorescent protein model marker, fluorescein isothiocyanate conjugated ovalbumin (OVA-FITC) was selected. The prepared MNs were immersed in an adequate solution of OVA-FITC (10 μg/mL dissolved in pH 7.2–7.5 PBS) for 10 min then removed and dried at 37 °C kept in a dark place. The porous MNs containing fluorescence were firmly placed on the rat’s back for 10 min and removed. Images were recorded separately using a fluorescence microscope (Zeiss Axio Observer Z1, Jena, Germany) with the same parameters and comparing the fluorescence intensity of porous MNs before and after application.

#### 2.4.5. Safety Evaluation

The Cell Counting Kit-8 (CCK-8) assay was performed to evaluate the toxicity of blank porous MNs on skin fibroblasts (Scheme S1). MNs were sterilized with 75% alcohol, then soaked three times in a sterile PBS solution to remove the alcohol, followed by UV irradiation for 2 h. Finally, MNs were pre-soaked in a culture medium for 24 h. The L-929 cells were inoculated in 96-well plates at a density of 1.0 × 10^4^ cells/well in Dulbecco’s modified Eagle medium (DMEM, containing 10% fetal bovine serum and 1% penicillin–streptomycin) and incubated at 37 °C and 5% CO_2_ for 24 h. Cells were cultured in MN pre-soaked medium for 24 h, while cells cultured in an un-soaked medium served as a negative control. Finally, the CCK-8 reagent was added and incubated for 2 h, and the relative cell viability was determined by measuring the absorbance at 450 nm with a microplate reader.

### 2.5. Colorimetric Glucose Detection

Using a UV–Vis spectrophotometer (Shimadzu UV-2700, Torrance, CA, USA), the absorption spectra was obtained from the reaction mixture consisting of 1 mL of glucose solution, 40 μL of GOx and HRP enzyme mixture, and 20 μL of TMB (15 mM anhydrous ethanol).

### 2.6. Image Analysis

A smartphone camera (iPhone 12mini, Cupertino, CA, USA) was used to capture the porous MN sensor images, while the lights directly above in the lab remained on to maintain consistency in the ambient lighting conditions. The smartphone and sensor were always kept at a fixed angle and distance using a stand. The captured images were further analyzed by ImageJ software (NIH, Bethesda, MD, USA). Specifically, a constant circular portion (diameter: 220 pixels) was selected for each detection area. Thereafter, the gray value was computed to obtain the relationship between the difference in gray (Δ Gray) and the glucose concentration in the detection area before and after the administration of the porous MN sensor. A digital colorimeter was used to obtain the RGB native values, and the relationship between the RGB values and the glucose concentration was established.

### 2.7. Glucose Colorimetric Detection with Porous Microneedle Sensor

Porous MN sensors were applied to agarose hydrogels containing different glucose concentrations (25–400 mg/dL) for 10 min, and color differences were observed. Meanwhile, for agarose gels with varying glucose concentrations, MN extraction mass was recorded, and RGB values and Δ Gray were obtained as described in Section 2.6.

### 2.8. In Vivo Performance of Porous Microneedle Sensor

The diabetic rat model was established using streptozotocin (STZ) induction, and a blood glucose meter (Yuwell 580) was chosen to monitor 3 μL tail vein blood glucose levels. The day prior, rats had their backs shaved. A porous MN sensor was firmly placed on the back for 10 min, during which time the sensor changed color and was eventually removed. Meanwhile, blood glucose levels were measured during the experiment. After that, image acquisition and analysis of the MN sensing layer were performed following Section 2.6.

To study skin healing after the insertion of MNs into rats’ skin, the rats’ backs were depilated. The MNs were pressed onto the back skin for 10 min and then removed. The skin was then photographed, and the healing process was monitored.

### 2.9. Statistical Analysis

The two groups were compared using the independent-samples t-test, and all results were expressed as mean ± standard deviation. GraphPad Prism 9.0.0 (GraphPad Software, Inc., La Jolla, CA, USA) was used for all statistical analyses, and differences were considered statistically significant at *p* < 0.05.

## 3. Results and Discussion

### 3.1. Characterization of Porous Microneedles

#### 3.1.1. Physical Characterization of Porous Microneedles

The porous MN morphology was characterized by microscopy (Figure 1B,C). The MNs have a white appearance and consist of 129 well-ordered needles with a conical columnar tip and a smooth homogeneous surface. The resulting needles measured 813.91 ± 13.42 μm in height and 345.54 ± 4.73 μm in base diameter. The SEM image of the interior of the needle tip as seen in Figure 1D indicates that the prepared porous MNs have internal pores. Figure 1E shows the pore size distribution using a mercury piezometer, yielding an average pore size of 264.63 nm. In the context of ISF sampling, the MN length reported in previous studies has ranged from 500 to 1000 μm [16,23,30]. The porous MN height of about 800 μm can penetrate through the epidermis (50–150 μm thick) and reach the dermis without damaging the subcutaneous blood vessels and nerves.

#### 3.1.2. In Vitro Extraction and Detection

The effect of different molecular weights of the porogen PEG on the uptake performance of porous MNs was evaluated, and the results are depicted in Figure 1F. Porous MNs absorbed the maximum in the agarose gel with a pure 10,000 molecular weight of PEG, extracting 63.13 ± 0.71 mg in 10 min, with a similar absorption for mixed PEG. The pure 4000 molecular weight absorbed the least because pores with smaller diameters are produced with the reduction in molecular weight [31]. Nevertheless, large-diameter pores can lead to poor mechanical properties, and porous MNs with PEG of pure 10,000 could not guarantee the penetration of each needle into the skin (Figure S1). Consequently, porous MNs with a PEG molecular weight of 4000 mixed with 10,000 (1:1, *v*/*v*) were chosen for subsequent experiments to maintain good absorption properties and mechanical strength.

Then, to validate the rapid sampling and collection of liquid by porous MNs using capillary action, this method was applied to agarose gels to obtain the mass of liquid absorbed at different time points as illustrated in Figure 1G. The results revealed that 34.57 ± 4.76 mg of simulated interstitial liquid could be rapidly absorbed within one minute. The amount of liquid absorbed increased over time, with no discernible difference after 10 min.

Porous MNs were pressed into agarose skin models containing varying concentrations of glucose, and the absorbance of the liquid recovered by porous MNs increased with the glucose content of the model, exhibiting a linear relationship as seen in Figure 1H. This indicates that porous MNs can be viably used to extract and detect biomolecules such as glucose in skin models.

#### 3.1.3. Penetration through Rat Skin

The investigations on the mechanical properties of porous MNs confirmed they were effective in penetrating the skin (Figure 1I,J). Following the insertion of porous MNs into the skin of fresh rat cadavers, visible pores were evident after trypan blue staining. H&E staining of skin sections after MN insertion revealed the formation of cone-shaped cylindrical pores within the skin.

#### 3.1.4. Protein Retention Test

The fluorescence intensity of the porous MNs with labeled with fluorescent protein was slightly lower than before application in rats, but there was no statistical significance (*p* > 0.5) as shown in Figure S2. Because of the safety of the fixed bioenzyme [32], there is no need to worry about damage to the skin, even if there is partial release. The subsequent in vitro and in vivo sensor application experiments in this study demonstrate the good performance of the sensor.

### 3.2. Colorimetric Glucose Detection

**Figure 2:**
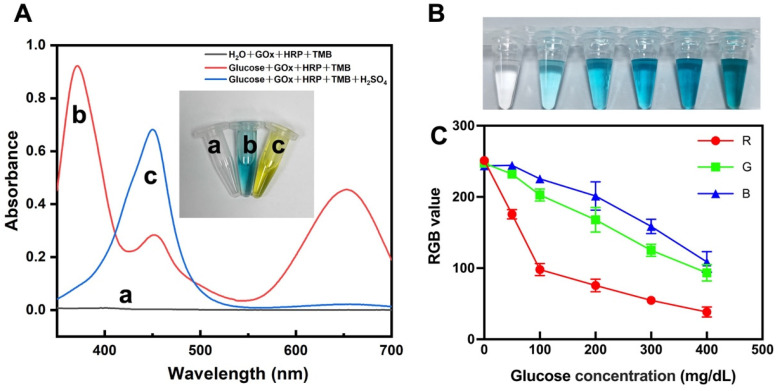
Glucose colorimetric assay. (**A**) UV–Vis absorption spectra of a: H_2_O + GOx + HRP + TMB, b: glucose + GOx + HRP + TMB, c: glucose + GOx + HRP + TMB + H_2_SO_4_ (the inserted image: photograph of solutions a, b, and c). (**B**) Photographs of varying concentrations of glucose reacting with enzymes and chromogenic agents. (**C**) RGB mean values of the image in (B) (n = 3).

The UV–Vis spectra of the three solutions are depicted in Figure 2A. They show that the TMB oxidation product (TMBox) has two absorption peaks at 365 nm and 650 nm, in contrast to the control group using pure water, which showed no color. The glucose solution + GOx + HRP + TMB reaction was terminated when H_2_SO_4_ was added to the solution, and the solution changed color to yellow with an absorption peak at 450 nm. The above results confirm that GOx + HRP can oxidize TMB in glucose solution, producing a significant color change. As shown in Figure 2B,C, a gradual deepening of the blue color was observed in glucose solutions with a concentration gradient (50–400 mg/dL), accompanied by a drop in RGB values, indicating the potential of the present colorimetric approach for glucose detection.

### 3.3. Optimization of Colorimetric Test Conditions

#### 3.3.1. Influence of Reaction Time

The reaction time affects the color intensity, so the effect of reaction time on the Δ Gray value was investigated. As seen in Figure S3A, the color intensity of the detection area increases with time, with a significant change in the first 10 min. Considering the need for rapid detection, the optimal reaction time of 10 min was chosen for the next experiments.

#### 3.3.2. Influence of Different Concentrations of the TMB

The effect of TMB concentration was examined for the glucose assay (Figure S3B). The TMB concentration of 3 mM was selected since the Δ Gray values decreased at concentrations below or above this level.

### 3.4. Glucose Colorimetric Detection with Porous Microneedle Sensor

**Figure 3:**
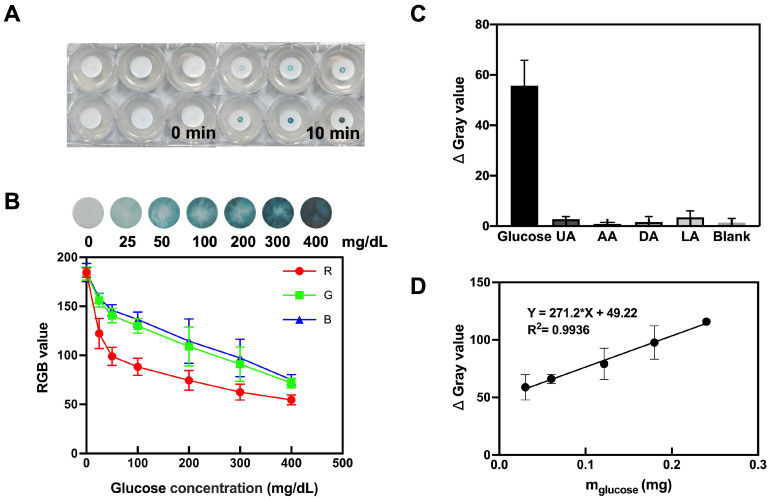
Colorimetric detection of glucose by porous MN sensor. (**A**) Images showing porous MN color change in hydrogels consisting of 1.4% agarose containing different concentrations of glucose. (**B**) Mean RGB values of the images (n = 3). (**C**) Porous MN sensor selectivity test (n = 3). (**D**) The calibration curve of the relationship between glucose content and Δ Gray was obtained by porous MN sensors in agarose gels with varying glucose concentrations (n = 3). R^2^ = 0.9936 as analyzed by simple linear regression.

In this study, the analytical performance of the filter-paper-based MN colorimetric technique for glucose detection was evaluated under optimal conditions. When porous MN sensors were applied to agarose hydrogels containing different glucose concentrations for 10 min, significant color differences were observed (Figure 3A,B). The analysis of the RGB values using ImageJ provides the corresponding quantitative information. As glucose levels increase, the sensor detection area becomes darker and the RGB values show a decline at distinct rates, i.e., red (185 to 55) > green (183 to 72) > blue (185 to 75). The resulting blue color visible to the naked eye can be used to determine glucose levels. Meanwhile, for agarose gels with different glucose concentrations, the calibration curve of the relationship between glucose content and Δ Gray was obtained by porous MN sensors, as shown in Figure 3D.

### 3.5. Selectivity

The porous MN sensor was inserted into glucose solutions, blank solutions, and solutions of interfering substances found in the blood, such as uric acid, ascorbic acid, dopamine, and lactic acid. Figure 3C displays the results, showing that the samples with interfering substances (uric acid (UA), ascorbic acid (AA), dopamine (DA), and lactic acid (LA)) had negligible color intensity compared to the glucose-containing samples. These findings validate that the porous MN sensor has good selectivity, offering the possibility of its application in complex biological samples.

### 3.6. In Vitro Detection Performance of Porous Microneedle Sensor

**Figure 4:**
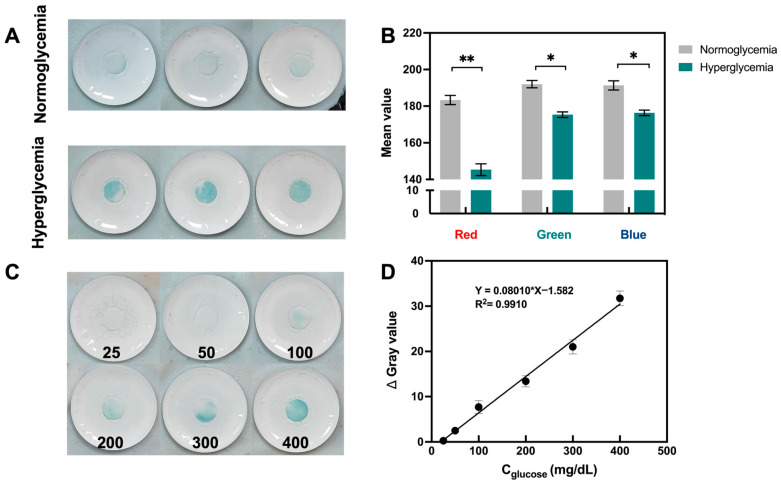
In vitro performance of porous MN sensor. (**A**) Photographs of porous MN sensor inserted in the skin of rats with normoglycemia and hyperglycemia, respectively. (**B**) RGB mean value of images in (**A**), results are expressed as mean ± SD (n = 3), * *p* < 0.05, ** *p* < 0.01. (**C**) Images of porous MN sensor insertion on rat skin with varying glucose concentrations (25–400 mg/dL). (**D**) Analytical curves for glucose detection by porous MN sensor (n = 3).

The in vitro performance of the porous MN sensor was investigated by examining isolated rat skin that had been treated with glucose solution. To allow for adequate glucose molecule penetration into the skin, isolated rat skin was immersed in a PBS solution equivalent to normoglycemic (100 mg/dL) and hyperglycemic (300 mg/dL) levels for 24 h. The sensor penetrated the treated rat skin and was held to ensure adequate extraction of the fluid. The color change to blue could be observed with the naked eye in the hyperglycemic group, while analysis of the captured images also showed a significant decrease in the mean RGB values (Figure 4A,B). Figure 4C,D shows that the Δ Gray values as a function of concentration on isolated rat skin with various glucose concentrations demonstrated strong linearity in the range of 25–400 mg/dL (R^2^ = 0.9910).

### 3.7. In Vivo Performance and Safety Assessment

**Figure 5:**
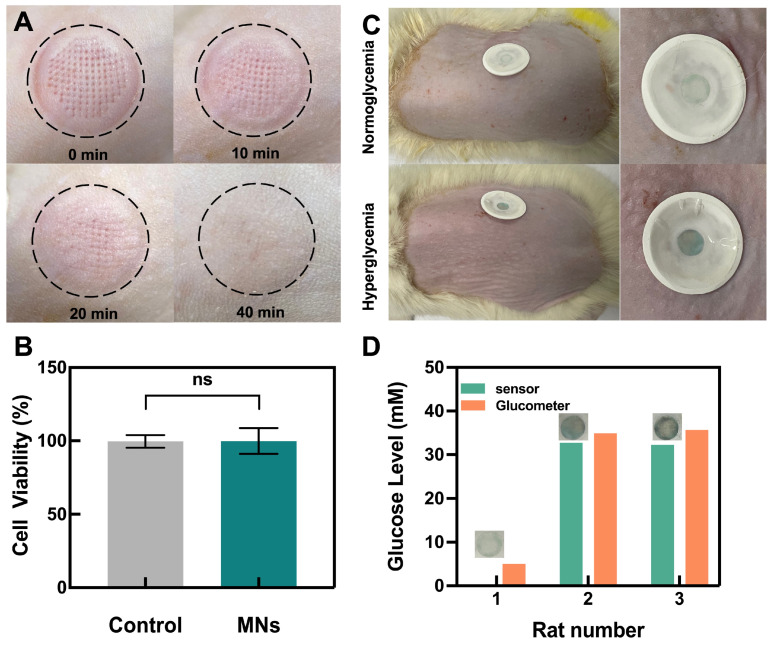
Performance of porous MN sensor in the rats and safety assessment. (**A**) Skin recovery after sensor insertion. (**B**) Cytocompatibility assay of porous MNs material (n = 3). (**C**) Photographs of glucose levels in rats as measured by porous MN sensor. (**D**) Glucose levels in ISF or blood glucose levels were measured in parallel by the porous MN sensor and a glucometer.

The safety of the porous MN sensor application was evaluated, as shown in Figure 5A. After inserting MNs into the skin of rats for 10 min, obvious holes were visible in the skin after the removal of the sensor. However, these holes gradually disappeared with time and almost entirely healed after 40 min, during which no obvious irritation occurred. The CCK-8 method was used to examine the cytocompatibility of the MN-making material on L-929 fibroblasts, as seen in Figure 5B. The cellular activity was ~100% in both the control and microneedle groups with no statistical difference, indicating that the material is biocompatible. 

Finally, the porous MN sensor was used in vivo to detect glucose in streptozotocin (STZ)-induced SD diabetic rats and healthy rats. When the sensor was removed after 10 min of application, the sensor remained colorless in the normal group, while it turned blue in the hyperglycemic group, indicating the feasibility of the porous MN sensor for detecting hyperglycemia in diabetic rats (Figure 5C). The agarose skin model was used to calculate the glucose concentration of the ISF from the measured Δ Gray, using the linear section of the calibration curve (Figure 3D). The calculated glucose concentration was then compared to readings from a commercial blood glucose meter by measuring blood glucose in rat tail veins (Figure 5D). All rats with hyperglycemia measurements from the porous MN sensor were found to have similar results when compared to those from the commercial glucose meter. The rats with normal glucose levels cannot be calculated because the Δ Gray value was below the limit of quantification. Meanwhile, MNs extracted 17.36 ± 1.98 mg of ISF from the rats in 10 min of the experiment. This quantity of extraction reaches the sensing layer by capillary action and is adequate to satisfy the filter paper’s absorption to saturation while allowing the porous MN sensor to extract enough ISF for subsequent biosensing.

Glucose levels were measured in both the blood and ISF of the animals, and the results indicated that the glucose level in the blood was slightly higher than that in ISF, consistent with the previous report [33]. Blood glucose levels and ISF glucose levels are generally well correlated. Previous studies have shown a time lag of 4–10 min in ISF glucose levels relative to blood glucose levels [34]. After measuring blood glucose in rats via the tail vein, photographs of the detection area were recorded when using the porous MN sensor immediately for 10 min, and then the two values were compared. The hyperglycemic rat model was chosen for the animal study, and the rats maintained high levels of blood glucose throughout the in vivo application experiment of the sensor.

In this study, PGMA porous MNs could be synthesized by UV polymerization in one step, and the enzymes used for the reaction were loaded into the porous matrix by immersion to make the reaction more efficient. In addition to in vitro, we further validated the sensor on animal subjects where the color change takes only 10 min.

Animal studies showed that the porous MNs can extract enough skin ISF utilizing the capillary force of the pores and that the rate of extraction and color change matches the requirements of the assay. According to the findings, the difference in color intensity produced by normoglycemia and hyperglycemia was apparent to the naked eye. Due to the difference in extraction volumes between the in vitro experiments and the rat studies, more research on the concentration of fixed enzymes as well as the choice and concentration of the chromogenic agent will be required for further glucose quantification in hyperglycemia.

## 4. Conclusions

In this study, the porous MN sensor was fabricated for minimally invasive sampling and colorimetric sensing of glucose in ISF. Biocompatible porous MNs were prepared that can successfully penetrate rat skin and, due to their internal pores, can extract sufficient ISF for analysis in a short time. Further, the enzymes integrated with chromogenic agents on the porous MN sensor led to in situ visual colorimetric detection of glucose. With the aid of a smartphone, the color intensity of the paper-based detection area was analyzed, resulting in the quantification of glucose levels. The usage of the porous MN sensor was successfully validated on rat models as well as on rat skin with varying glucose levels. The device is easily portable and requires only finger pressure for obtaining glucose levels. In the future, the creation of an integrated, minimally invasive sampling and sensing platform can be further extended to detect other biomarkers in ISF, enhancing patient compliance and detection efficiency.

## Data Availability

All data are available in the manuscript and Supplementary Materials.

## Supplementary Materials

The following supporting information can be downloaded at: https://www.mdpi.com/article/10.3390/bios13050537/s1: Figure S1: Skin penetration in rats with porous MNs prepared from the porogen PEG with a molecular weight of 10,000; Figure S2. Protein retention test. (A) Fluorescence microscopy images before and after the application of porous MNs on rats. (B) Fluorescence intensity of porous MNs on rats before and after application (n = 3); Figure S3. Optimization of assay conditions. (A) Influence of reaction time on glucose detection (n = 3). (B) Influence of different concentrations of the chromogenic agent TMB on glucose detection (n = 3); Scheme S1 Schematic illustration of the test for porous MNs cytotoxicity.
