# Supplementary material for: Porous Colorimetric Microneedles for Minimally Invasive Rapid Glucose Sampling and Sensing in Skin Interstitial Fluid
Source: Biosensors (Basel). 2023 May 10;13(5):537. doi: 10.3390/bios13050537 (PMC10216140; doi:10.3390/bios13050537)
Supplement: Supplementary file 1 [file biosensors-13-00537-s001.zip › biosensors-2324517-supplementary.pdf]

biosensors

*Supplementary Materials*

# Porous Colorimetric Microneedles for Minimally Invasive Rapid Glucose Sampling and Sensing in Skin Interstitial Fluid

**Qingya Zeng [1], Mengxin Xu [1], Weilun Hu [1], Wenyu Cao [1], Yujie Zhan [1], Yuxin Zhang [2], Qingqing Wang [1,3] and Tao Ma [1,3,*]**

1 School of Pharmacy, Bengbu Medical College, Bengbu 233030, China
2 School of Laboratory Medicine, Bengbu Medical College, Bengbu 233030, China
3 Anhui Engineering Technology Research Center of Biochemical Pharmaceutical, Bengbu Medical College, Bengbu 233030, China
* Correspondence: matao1992@foxmail.com

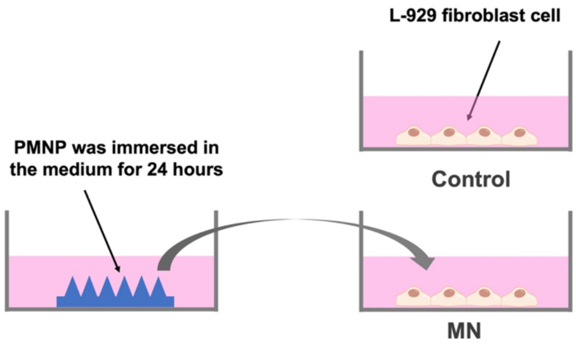


**Scheme S1.** Schematic illustration of the test for porous MNs cytotoxicity.

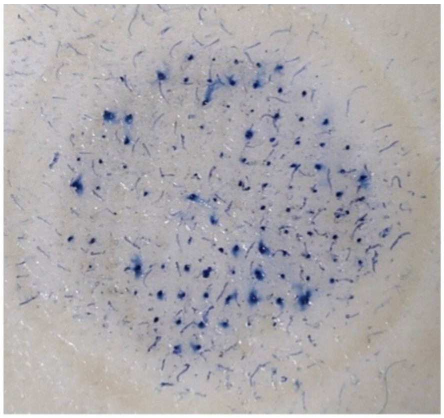

**Figure S1.** Skin penetration in rats with porous MNs prepared from the porogen PEG with a molecular weight of 10,000.

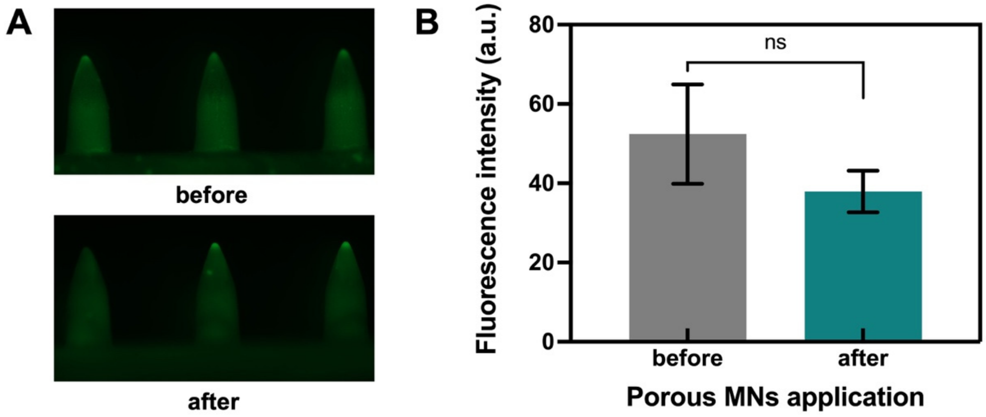


**Figure S2.** Protein retention test. (A) Fluorescence microscopy images before and after the application of porous MNs on rats. (B) Fluorescence intensity of porous MNs on rats before and after application (n=3).

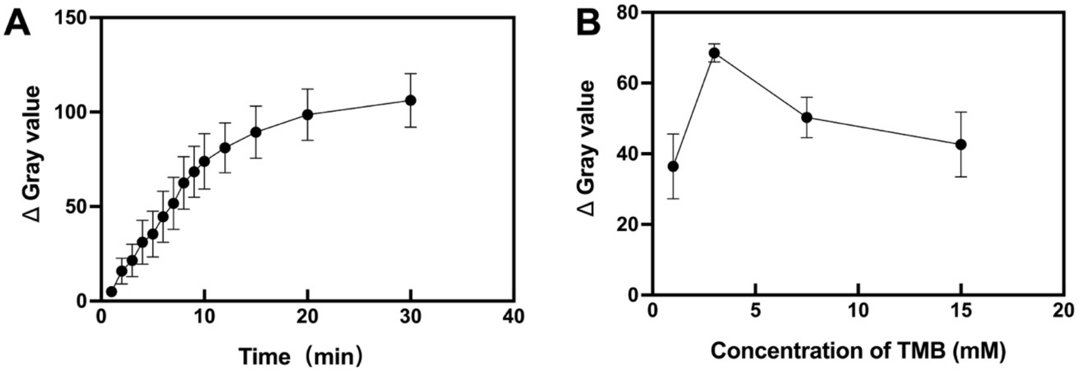


**Figure S3.** Optimization of assay conditions. (A) Influence of reaction time on glucose detection (n=3). (B) Influence of different concentrations of the chromogenic agent TMB on glucose detection (n=3).
